# Optimized Quasi-Optical Mode Converter for *TE*_33,12_ in 210 GHz Gyrotron

**DOI:** 10.3390/mi16030308

**Published:** 2025-03-06

**Authors:** Hamid Sharif, Muhammad Haris Jamil, Wenlong He

**Affiliations:** College of Electronics and Information Engineering, Shenzhen University, Shenzhen 518060, China; harisjamil07@gmail.com (M.H.J.); wenlong.he@szu.edu.cn (W.H.)

**Keywords:** quasi-optical (QO) mode converter, gyrotron, continuous wave (CW), Denisov-type launcher

## Abstract

This article discusses the design of a high-performance quasi-optical mode converter for the $TE_{33,12}$ mode at 210 GHz. The conversion process is challenging due to a caustic-to-cavity radius ratio of approximately 0.41. The mode converter employs an optimized dimpled wall launcher, analyzed using coupling mode theory with twenty-five coupled modes, compared to the usual nine modes and optimized reflector systems, to effectively address the conversion challenge.Electromagnetic field analysis within the launcher wall was optimized using MATLAB R2021b. The radiation fields from the launcher were analyzed in free space using Gaussian optics and vector diffraction theory. The mirror system consists of a quasi-elliptical mirror, an elliptical mirror, and phase-corrected parabolic mirrors. Following phase correction, the output window achieved a scalar Gaussian mode content of 99.0% and a vector Gaussian mode content of 97.4%.

## 1. Introduction

Gyrotrons are powerful devices that generate high-frequency microwaves and millimeter waves for applications like plasma heating, material processing, and advanced radar systems. They often operate in complex high-order transverse electric (TE) modes, which can be difficult to use. To address this, gyrotrons employ a quasi-optical (QO) mode converter, which transforms these high-order modes into more manageable, Gaussian-like beams. This conversion facilitates easier transmission and focusing, enhancing their suitability for various applications [1,2,3,4]. QO mode converters typically consist of a launcher that generates a helically symmetric wave and a mirror system that refines the wave into a Gaussian beam [5]. The performance of these converters is assessed by their mode conversion efficiency, beam quality, and tolerance to alignment errors, which are particularly important in high-power environments. The principle of QO mode conversion is based on the phase-space transformation of electromagnetic fields. The launcher creates a helically symmetric wave from the high-order mode, which is then refined through parabolic or elliptical mirrors and phase correction techniques [6,7,8], ultimately generating the desired beam profile. The design of these converters is often based on analytical techniques that calculate the exact field patterns required for efficient transformation. The QO mode converter is divided into two categories according to the launcher design: Vlasov-type and Denisov-type converters. The Vlasov-type converter features a simpler structure but encounters significant diffraction losses of approximately 15% to 20%, which are deemed unacceptable for high-power gyrotrons [9,10,11]. Denisov et al. proposed an enhanced launcher design focused on reducing diffraction losses and refining the dimensions of the focusing mirror system [3].

In this paper, we investigate a Denisov-type quasi-optical (QO) mode converter designed for the $TE_{33,12}$ mode operating at 210 GHz in a gyrotron. The $TE_{33,12}$ mode, with a caustic-to-cavity radius ratio of approximately 0.41, presents significant challenges for efficient conversion into a near-Gaussian beam distribution using a dimpled-wall Denisov launcher. These challenges arise because third-harmonic perturbations are ineffective in coupling modes with a first-mode index difference of three, thereby complicating the mode conversion process. A similar challenge is observed in the $TE_{22,8}$ mode, which has a comparable caustic-to-cavity radius ratio of about 0.4178, as reported in [12]. In contrast, conventional gyrotrons operating at frequencies such as 110 GHz [13,14], 140 GHz [15,16,17,18], and 170 GHz [19], with caustic-to-cavity radius ratios closer to 50%, demonstrate more efficient mode conversion. For these gyrotrons, the waveguide mode’s ray representation forms a nearly closed triangular pattern in the cross-section of the launcher, facilitating Gaussian-like beam generation. The launcher, designed using coupling mode theory, incorporates a periodic spiral structure. It typically combines nine modes with distinct relative power distributions to achieve an approximation of a Gaussian-like distribution [20,21]. It has been shown that the primary mode couples with multiple other modes under perturbation, particularly those with similar eigenvalues, which carry significant energy.

In this article, we employ coupled mode theory to superimpose 9 modes, along with an additional 16 modes for analysis. Nine coupling modes are utilized to approximate a Gaussian-like distribution, yielding a total output power of 96.936% from the waveguide. In contrast, increasing the number of coupling modes to 25 raises the total output power to 99.958%, significantly improving the efficiency and bringing it much closer to the input power. This results in a field distribution that more closely matches theoretical predictions. The inclusion of these extra modes increases the peak amplitude of the Gaussian wall current distribution and reduces side-lobe levels in power. Thus, the impact of these minor modes is substantial and must be accounted for when calculating the field distribution along the waveguide wall. Secondly, the $TE_{33,12}$ mode at 210 GHz, with a caustic-to-cavity radius ratio of about 0.41, creates significant difficulty in converting the cavity mode into a nearly Gaussian beam and achieving well-focused fields at the aperture, which are essential for reducing diffraction at the launcher’s edges. To deal with this problem, we design an optimized system mirror which mitigates the effects of the launcher and provides a well-focused beam at the output window. The mirror system includes a quasi-optical mirror, an elliptical mirror, and a parabolic mirror.

The initial dimensional parameters of the QO mode launcher are determined through a parametric evaluation using geometric optics theory [3]. These parameters are then input into the FEKO simulation tool (Altair Simulation Products Version 2021.1), which generates optimized inner-radius profiles for the mode launcher to ensure maximum performance (Section 2). A mirror section (Section 3) is then integrated into the mode launcher to form the complete mode converter. The findings are then analyzed in Section 4, with conclusions presented in Section 5.

## 2. Design of Launcher

The Denisov radiator achieves a periodic distribution of wall current by generating spiral ripples on the radiator wall. A minor disturbance alters the reflection direction of rays on the waveguide wall, causing them to converge. As a result, a Gaussian beam can be generated by combining particular waveguide modes, each with consistent amplitudes and relative phases. This provides a Gaussian profile with the desired field intensity on the wall of the launcher. This profile is converted from the power of the incident TE mode to satellite modes. Equation (1) provides an approximate description of the field distribution in two dimensions [22].

(1) $$\begin{array}{c} f\left(\phi ,z\right)=\left(1+\frac{1}{2}\exp\left(\frac{j\pi \phi }{\theta }\right)+\frac{1}{2}\exp\left(-\frac{j\pi \phi }{\theta }\right)\right)\left(1+\frac{1}{2}\exp\left(\frac{j2\pi z}{L}\right)+\frac{1}{2}\exp\left(-\frac{j2\pi z}{L}\right)\right) \end{array}$$

This function illustrates that the creation of a Gaussian beam stems from the interference of satellite waveguide modes in both the longitudinal and radial directions. This process must adhere to the following criteria:

(2) $$\begin{array}{cc} \Delta \beta & =\pm \frac{2\pi }{L_{c}},l=\Delta m=\pm \frac{\pi }{\theta },\cos\theta =\frac{m}{X_{mn}},\theta =\arccos=\frac{m}{X_{mn}} \end{array}$$

Here, *m* and *n* represent the azimuthal and longitudinal mode indices, respectively, $X_{mn}$ represents the root of the derivative of the Bessel function, $L_{c}$ denotes the launcher’s cut length, and $R_{c}$ and *R* stand for the caustic and launcher radii, respectively. The launcher features a tapered dimpled-wall design, implemented to prevent parasitic oscillations. To excite the desired modes, the inner wall features a two-tier perturbation approach, defined by the following equation [23]:

(3) $$\begin{array}{c} R\left(\phi ,z\right)=R_{0}+\alpha z+\delta_{1}\left(z\right).\cos\left(\Delta \beta_{1}z-l_{1}\phi \right)+\delta_{2}\left(z\right).\cos\left(\Delta \beta_{2}.z-l_{2}\phi \right) \end{array}$$

**Figure 1:**
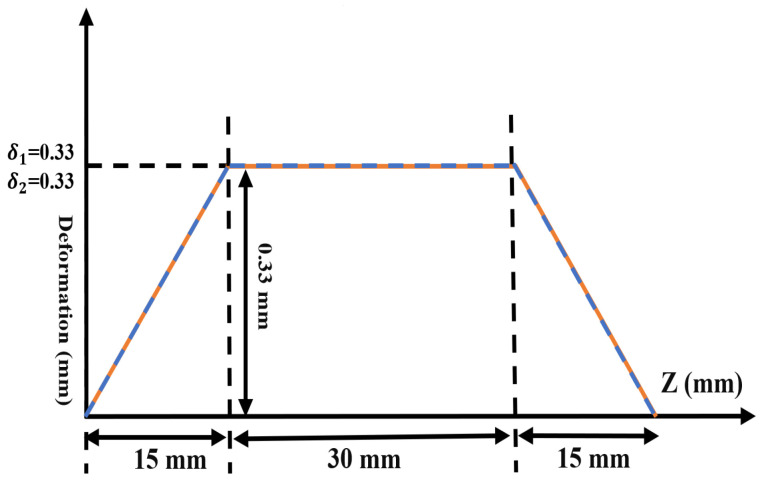
Waveguide wall disturbance curve distribution of Denisov radiator.

In this context, *R* denotes the average radius, α represents the taper slope (α=0.00004), and $\delta_{1}$ and $\delta_{2}$ indicate the disturbance magnitudes, which are depicted in Figure 1 and are fine-tuned to create a Gaussian distribution. ϕ stands for the azimuthal angle. $l_{1}$, $l_{2}$, $\Delta \beta_{1}$$\left(\Delta \beta_{1}=181.8813\right)$, and $\Delta \beta_{2}$$\left(\Delta \beta_{1}=46.6881\right)$ are defined as follows:

(4) $$\begin{array}{cc} l_{1} & =\pm (m_{1}-m2)=\pm 1 \end{array}$$

(5) $$\begin{array}{cc} l_{2} & =\pm \left(m_{1}-m2\right)=\pm \Delta m_{2} \end{array}$$

(6) $$\begin{array}{cc} \Delta \beta_{1} & =\frac{1}{2}\left(\beta_{m-1,n}-\beta_{m+1,n}\right)=\frac{1}{2}\left[\sqrt{k_{0}^{2}-{\left(\frac{X_{m,n}}{R_{0}+\alpha z}\right)}^{2}}-\sqrt{k_{0}^{2}-{\left(\frac{X_{m,n}}{R_{0}+\alpha z}\right)}^{2}}\right] \end{array}$$

(7) $$\begin{array}{cc} \Delta \beta_{2} & =\frac{1}{2}\left(\beta_{m-\Delta m,\Delta n}-\beta_{m+\Delta m,\Delta n}\right)=\frac{1}{2}\left[\sqrt{k_{0}^{2}-{\left(\frac{X_{m,n}}{R_{0}+\alpha z}\right)}^{2}}-\sqrt{k_{0}^{2}-{\left(\frac{X_{m,n}}{R_{0}+\alpha z}\right)}^{2}}\right] \end{array}$$

The design goal is to couple the energy of the $TE_{33,12}$ mode to each satellite mode in an appropriate proportion. However, when coupling occurs, a π/2 phase is introduced to the satellite mode. Each mode needs to propagate over a certain distance to match the phase and achieve an approximately Gaussian surface current distribution. That is to say, the Denisov radiator can be considered to be composed of a disturbance section, a phase-matching section, and a radiation notch.

Mode coupling theory (CWT, Coupled Wave Theory) can be used to analyze the coupling effect of $TE_{33,12}$ to the satellite mode under different disturbance parameter conditions. Generally, the equations that describe the coupling of forward-propagating waves are expressed as follows:

(8) $$\begin{array}{c} d\left(A_{i}\right)/dz=-j\beta_{i}A_{i}+\sum_{k}C_{ip}A_{k} \end{array}$$

We concentrate solely on the TE mode. The coefficient $C_{ip}$ is formulated as follows [13]:

(9) $$C_{ip}=\frac{j\delta }{2R\sqrt{k_{zi}k_{zp}}\sqrt{{x^{\prime }}_{m1p1}^{2}-m_{1}^{2}}\sqrt{{x^{\prime }}_{m2p2}^{2}-m_{2}^{2}}}\left[\frac{{x^{\prime }}_{m1p1}^{2}}{R^{2}}\left({x^{\prime }}_{m1p1}^{2}-m_{1}m_{2}\right)\pm \Delta \beta k_{zp}\epsilon m_{1}m_{2}\right]\exp^{\pm \Delta \beta z}$$

With the help of the CMT method, the coupling from the main mode ($TE_{33,12}$) to other modes can be obtained from the waveguide size and disturbance parameters, and then the surface current distribution of the inner wall of the circular waveguide can be obtained.

**Figure 2:**
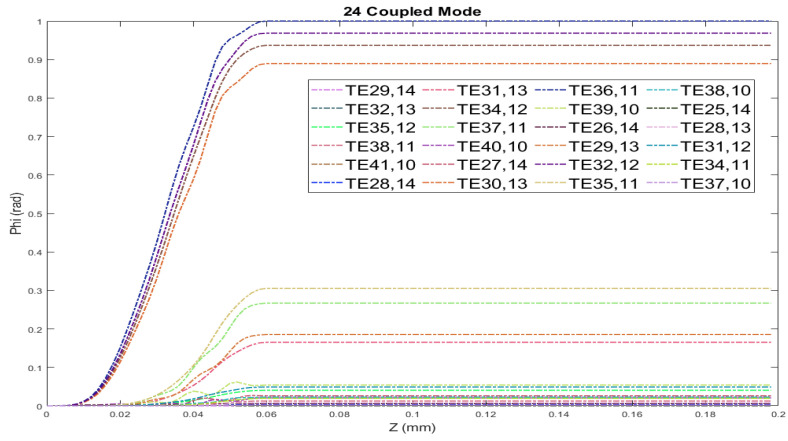
Variation in relative power coefficients for the $TE_{33,12}$ mode along the z-axis in the launcher, depicting coupling with 24 modes.

**Figure 3:**
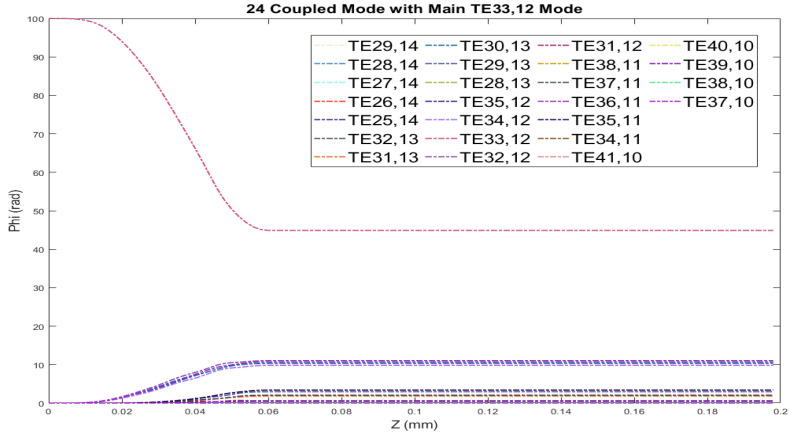
Variation in relative power coefficients for the $TE_{33,12}$ mode along the z-axis in the launcher, depicting coupling with 25 modes along with the main mode.

The waveguide mode converter is examined using coupled mode theory, and the radiated fields are computed using the vector diffraction integral from the waveguide cut. The $TE_{33,12}$ mode couples into twenty-four satellite modes along the z-axis due to the wall perturbations depicted in Figure 2. This figure illustrates the twenty-four coupled modes and their relative power along the z-axis, excluding the main mode. In this article, we consider 25 coupling modes, including the main mode, which is input into the waveguide depicted in Figure 3, from which it is evident that a pure $TE_{33,12}$ mode is initially input and converted into a satellite mode gradually. In coupled mode theory, the superposition of 25 modes generates a Gaussian-like mode, leading to a calculated field distribution that closely matches theoretical predictions. Table 1 presents the relative power of each mode at the waveguides output for the case of 25 coupled modes.

Traditionally, nine coupling modes are utilized to achieve a Gaussian-like distribution, resulting in an output power of just 96.936% from the waveguide. By increasing the number of coupling modes to 25, the total output power improves to 99.958%, bringing it significantly closer to the input power. These additional modes enhance the peak amplitude of the Gaussian wall current distribution and lower the side-lobe levels. Thus, their contribution to the superposed field is substantial and must be taken into account when calculating the field distribution along the waveguide wall using coupled mode theory. This optimization effectively aids in designing a launcher for the $TE_{33,12}$ mode at 210 GHz for a gyrotron.

**Figure 4:**
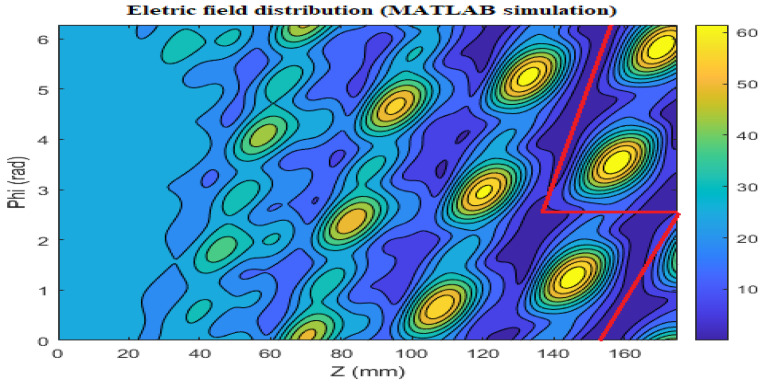
Electric field distribution on the launcher wall for the $TE_{33,12}$ mode at 210 GHz, simulated with MATLAB.

**Figure 5:**
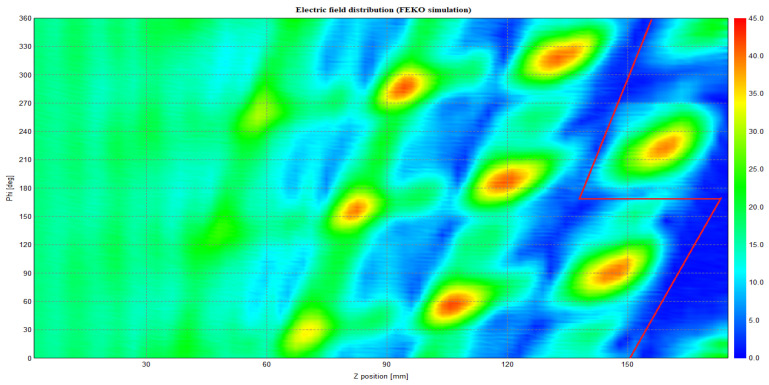
Electric field distribution on the launcher wall for the $TE_{33,12}$ mode at 210 GHz, simulated with FEKO.

Figure 4 and Figure 5 illustrate the current distribution on the waveguide wall of the Denisov radiator, as obtained from MATLAB R2021b and the FEKO simulation tool (Altair Simulation Products Version 2021.1), respectively. They illustrate, following the controlled disturbance of the waveguide’s inner wall, that the TE33,12 mode gradually develops into a high-quality Gaussian beam spot. This formation enhances the directional radiation and transmission of wave energy in space. The fields emanate from the edge of the launcher and are directed toward the mirror system.

## 3. Optical Path Design of Three-Mirror System

**Figure 6:**
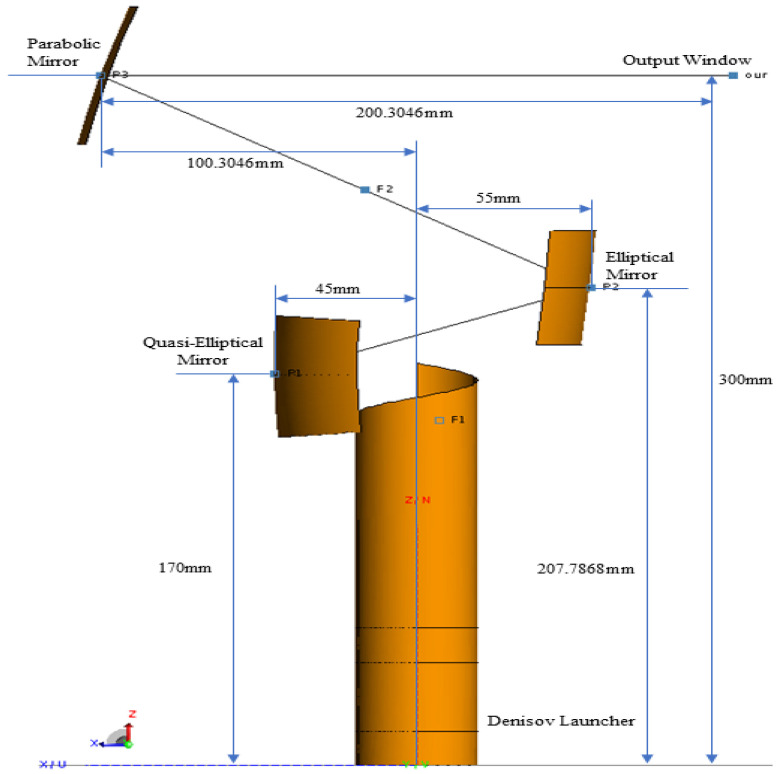
Optical path layout of the QO mode converter system.

A QO mode converter combines a launcher with multiple beam-forming mirrors to correct significant discrepancies between the emitted field and the intended distribution. To achieve the desired paraxial beam structure, the mirror system typically comprises quasi-parabolic or quasi-elliptical mirrors, complemented by one or two custom-designed mirrors. When integrated into the gyrotron, this mirror system shapes the spatial field distribution at the output window surface. The mirror system described in this article for beam shaping includes a quasi-elliptical mirror, an elliptical mirror, and a parabolic mirror, integrated with a phase correction function. Together, they guide the beam to create an accurate pattern on the gyrotron output window. Figure 6 illustrates the overall optical path layout of the Denisov transmitter and the mirror system, which comprises three reflective surfaces.

The quasi-elliptical primary mirror of the QO mode converter reconverges the beam in the horizontal plane. By placing its secondary focus closer to the z-axis, rather than at the far exit surface, the lateral dimensions of the primary reflector can be substantially reduced. The position of the quasi-elliptical mirror is determined by substituting Equation (10) into Equations (11) and (12):

(10) $$\begin{array}{c} l\left(\phi \right)=\frac{8R_{c}l_{2}\cos\phi -4\phi^{2}R_{c}^{2}-4\phi R_{c}^{2}\pi +8\phi R_{c}l_{g}-\pi^{2}R_{c}^{2}+4\pi R_{c}l_{g}+8l_{1}l_{g}-8l_{1}l_{2}}{8l_{2}\sin\phi +8\phi R_{c}+4\pi R_{c}+4\pi R_{c}+8l_{g}} \end{array}$$

(11) $$x\left(\phi \right)=R_{c}\cos\phi -l\left(\phi \right)\sin\phi$$

(12) $$y\left(\phi \right)=R_{c}\sin\phi -l\left(\phi \right)\cos\phi$$

The elliptical reflector also reconverges the beam in the horizontal plane. Its initial focus coincides with the secondary focus of the primary quasi-elliptical mirror, while its secondary focus is positioned at the distant exit surface. Moreover, the elliptical reflector operates like a zoom lens with an adjustable focal length along the z-axis. The parabolic lens converges the beam in the vertical dimension, with its focus centered on the exit surface. Additionally, future designs will incorporate a phase correction surface function in the parabolic lens.

## 4. Analysis of Calculation Results

**Figure 7:**
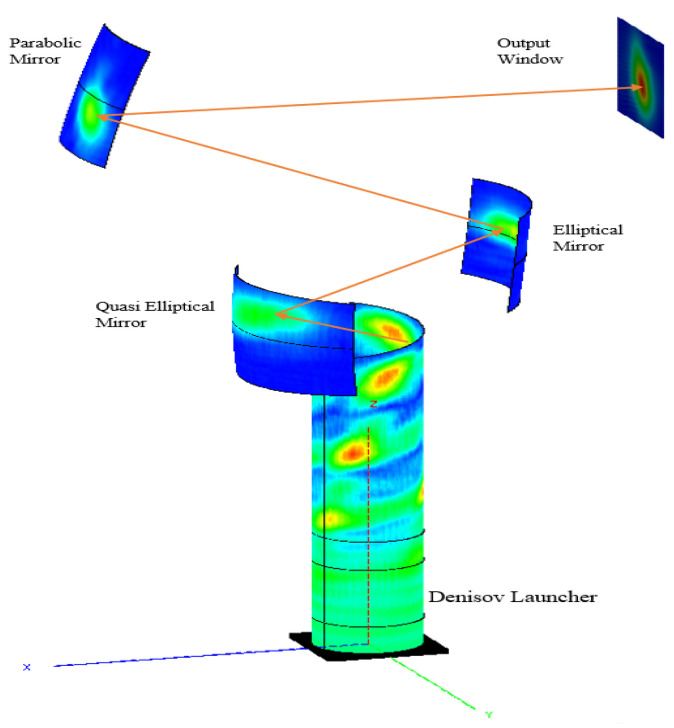
Surface current distribution on surface of launcher and mirror system.

The surface current distribution and the amplitude distribution of the reflected fields from all mirror levels exhibit a Gaussian-like pattern, indicating consistent directionality throughout the beam propagation process, as illustrated in Figure 7.

**Figure 8:**
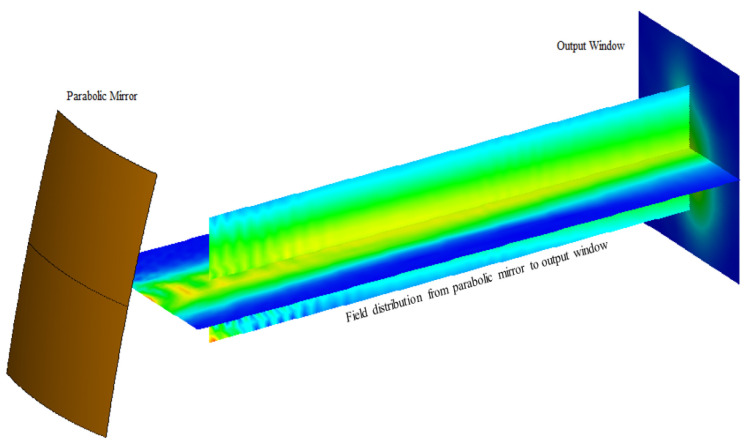
Beam propagation effect near the exit surface.

After accurately determining the radiation field distribution of the transmitter, the beam convergence effect of the radiator system is thoroughly calculated and analyzed. The analysis begins with the full-wave radiation field generated by the transmitter, followed by the calculation of the reflected field at the quasi-elliptical mirror. The incident field on the surface of the secondary mirror is recorded first. This is followed by the calculation of the exit field from the second-level mirror, and subsequently, the reflected field through the third-level mirror is determined, along with the field distribution on the exit surface. Figure 8 displays the beam propagation effect near the exit surface, as generated by the FEKO simulation.

**Figure 9:**
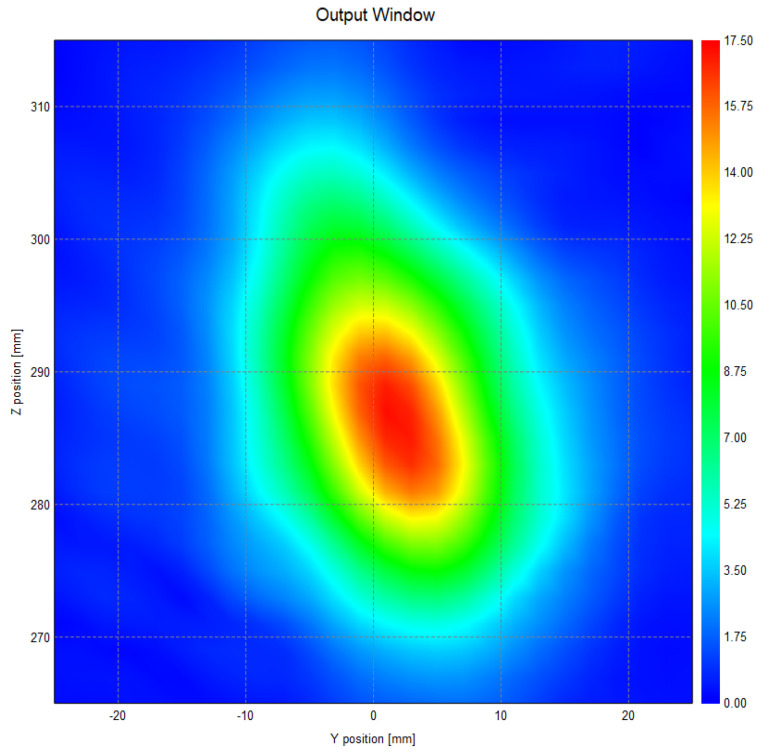
Distribution of electric fields at the exit surface.

**Figure 10:**
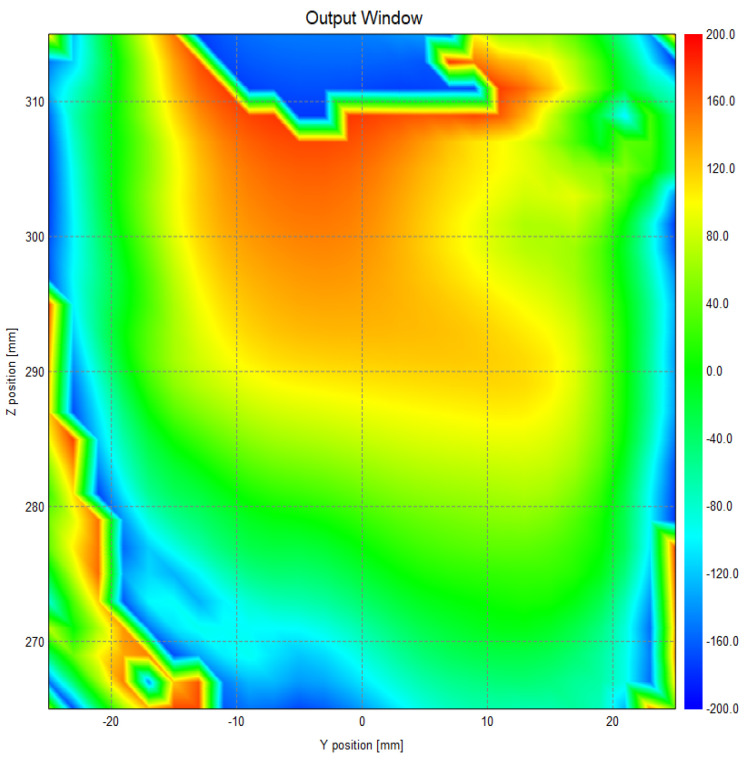
Phases at the exit surface.

Figure 9 and Figure 10 show the field and phase distributions at the exit surface. The results confirm that the beam converges as expected on the exit surface, with no observable aberrations in beam propagation and the formation of a well-defined beam waist at the exit surface. The distribution of surface currents, as well as the amplitude distribution of the reflected fields across all mirror levels, exhibits a Gaussian-like profile. This observation highlights the effective maintenance of beam directionality throughout the propagation process.

The performance of a wave beam is typically evaluated using its correlation coefficient with the ideal fundamental Gaussian mode ($TEM_{00}$ mode). This correlation can be expressed in two ways: the scalar correlation coefficient $\eta_{s}$, which takes into account only the amplitude, and the vector correlation coefficient $\eta_{v}$, which incorporates both amplitude and phase. These coefficients are defined as follows [24]:

(13) $$\eta_{s}=\frac{\int_{s}|u_{i}\left|.\right|u_{g}|ds}{\sqrt{\int_{s}|u_{i}{|}^{2}.ds\int_{s}{\left|u_{g}\right|}^{2}.ds}}$$

(14) $$\eta_{v}=\frac{\int_{s}|u_{i}\left|.\right|u_{g}|\exp\left[j\left(\phi_{i}-\phi_{g}\right)\right]ds*\int_{s}|u_{i}\left|.\right|u_{g}|\exp\left[j\left(\phi_{i}-\phi_{g}\right)\right]ds}{\sqrt{\int_{s}|u_{i}{|}^{2}.ds\int_{s}{\left|u_{g}\right|}^{2}.ds}}$$

**Figure 11:**
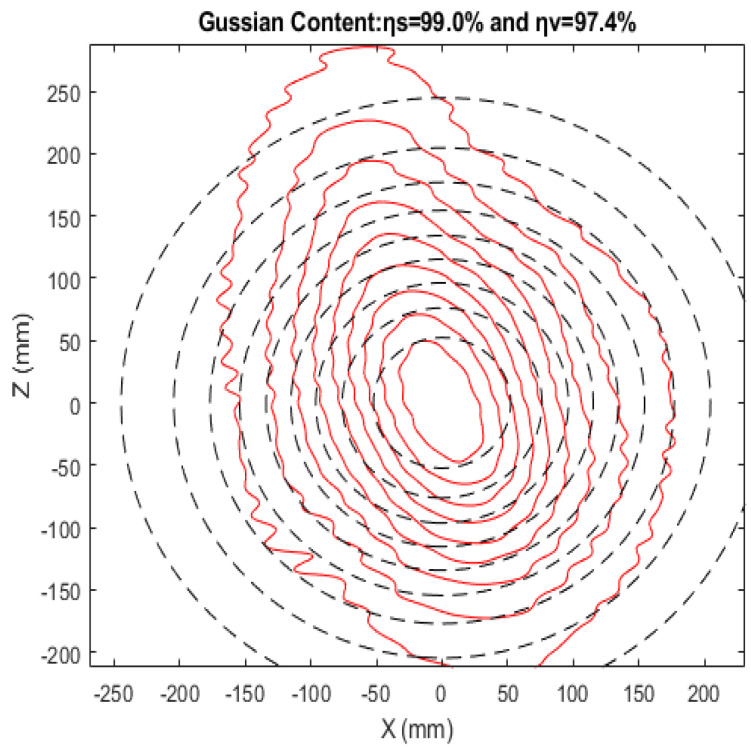
The solid line represents the wave-beam mode, while the dashed lines indicate the ideal Gaussian distribution.

In this context, $|u_{1}|\exp\left(j\phi_{1}\right)$ denotes an ideal fundamental Gaussian beam, and $|u_{2}|\exp\left(j\phi_{2}\right)$ represents the wave beam’s field distribution. At 100% mode purity, with no high-order modes present, $\eta_{s}$ and $\eta_{v}$ both take a value of 1. Although the dimpled-wall launcher efficiently converts cavity modes into nearly Gaussian beams, a few high-order modes still remain. In our model, the scalar Gaussian mode content ($\eta_{s}$) and the vector Gaussian mode content ($\eta_{v}$) at the output window are calculated to be 99.0% and 97.4%, respectively, for $TE_{33,12}$ at 210 GHz. Figure 11 illustrates the field contours at the output window, highlighting the beam’s exceptional Gaussian purity and the significant reduction in high-order modes.

## 5. Conclusions

A quasi-optical mode converter based on the Denisov design has been developed to convert the $TE_{33,12}$ mode at 210 GHz into a Gaussian beam. The converter incorporates an enhanced dimpled-wall launcher and three optimized reflectors designed to overcome the launcher’s limitations, which are characterized by a caustic-to-cavity radius ratio of 0.41. Utilizing 25 modes, the launcher produces a well-focused field at its aperture. The mirror system operates efficiently with a caustic-to-cavity radius ratio of less than 50%. This system consists of a quasi-elliptical mirror, an elliptical mirror, and a phase-correcting parabolic mirror. After phase correction, the output window achieves a scalar Gaussian mode content of 99.0% and a vector Gaussian mode content of 97.4%.

## Figures and Tables

**Table 1:** Relative power distribution of the operating mode and 25 coupled modes, including the main mode, at the waveguide output.

	Azimuthal Bunching →				
**Axial bunching**→	$TE_{29,14}$ 0.00139%	$TE_{32,13}$ 0.047%	$TE_{35,12}$ 0.287%	$TE_{38,11}$ 0.218%	$TE_{41,10}$ 0.004%
	$TE_{28,14}$ 0.0654%	$TE_{31,13}$ 1.822%	$TE_{34,12}$ 9.813%	$TE_{37,11}$ 2.046%	$TE_{40,10}$ 0.089%
	$TE_{27,14}$ 0.447%	$TE_{30,13}$ 10.339%	$TE_{33,12}$ 44.881%	$TE_{36,11}$ 10.686%	$TE_{39,10}$ 0.540%
	$TE_{26,14}$ 0.140%	$TE_{29,13}$ 2.945%	$TE_{32,12}$ 11.034%	$TE_{35,11}$ 3.370%	$TE_{38,10}$ 0.067%
	$TE_{25,14}$ 0.016%	$TE_{28,13}$ 0.228%	$TE_{31,12}$ 0.598%	$TE_{34,11}$ 0.252%	$TE_{37,10}$ 0.023%

## Data Availability

The data that support the findings of this study are available from “the corresponding author” upon reasonable request.
